# Scaling-up PrEP Delivery in Sub-Saharan Africa: What Can We Learn from the Scale-up of ART?

**DOI:** 10.1007/s11904-019-00437-6

**Published:** 2019-02-22

**Authors:** Gabrielle O’Malley, Gena Barnabee, Kenneth Mugwanya

**Affiliations:** Department of Global Health, University of Washington, Seattle, WA USA

**Keywords:** Pre-exposure prophylaxis (PrEP), Scaling-up, PrEP implementation, Sub-Saharan Africa, HIV/AIDS, Anagogical reasoning

## Abstract

**Purpose of Review:**

Clinical trials have found that PrEP is highly effective in reducing risk of HIV acquisition across types of exposure, gender, PrEP regimens, and dosing schemes. Evidence is urgently needed to inform scale-up of PrEP to meet the ambitious WHO/UNAIDS prevention target of 3,000,000 individuals on PrEP by 2020.

**Recent Findings:**

Successful models of delivering HIV services at scale evolved from years of formal research and programmatic evidence. These efforts produced lessons-learned relevant for scaling-up PrEP delivery, including the importance of streamlining laboratory tests, expanding prescription and management authority, differentiating medication access points, and reducing stigma and barriers of parental consent for PrEP uptake. Further research is especially needed in areas differentiating PrEP from ART delivery, including repeat HIV testing to ensure HIV negative status and defining and measuring prevention-effective adherence.

**Summary:**

Evidence from 15 years of ART scale-up could immediately inform a public health approach to PrEP delivery.

## Introduction

The scale-up of antiretroviral therapy (ART) over the past 20 years is one of the biggest public health achievements of the century [1, 2]. Although highly active antiretroviral therapy became widely available in 1996 in high-income countries, access was minimal in sub-Saharan Africa (SSA) where the HIV/AIDS burden was highest [3]. In 2000, approximately 6600 HIV-infected individuals were on treatment in SSA as compared to 664,000 in Europe and the Americas [4]. International advocacy efforts for universal ART access gained steady momentum with landmark initiatives such as the World Health Organization (WHO) 3 by 5 initiative, the establishment of the global fund to fight HIV, tuberculosis and malaria, and the President’s Emergency Plan for AIDS Relief (PEPFAR). Even amidst these calls to action and significant dedication of funds, reservations persisted about the promotion of ART in Africa, centering around the cost and complexity of treatment, weak African health systems, human resource constraints, and likely poor adherence to treatment [5, 6]. Partially in response to these concerns, initial global investments in ART delivery were focused on “centers of excellence,” mainly in tertiary hospitals located in major cities. While treatment for people living with HIV/AIDS (PLWHA) in SSA did increase to 2,950,000 by 2008 [7], this figure represented only 44% of those needing treatment at that time. It became increasingly apparent that additional approaches would be necessary to truly achieve ART access at scale.

In 2010, the UNAIDS Secretariat and the WHO launched “Treatment 2.0,” an initiative to simplify and optimize ART delivery based on the previous decade of programmatic and clinical evidence and experience [8]. Since then, global and national scale-up efforts have frequently moved ahead of evidence provided by formal implementation trials [9, 10]. By 2017, there were approximately 15,000,000 people in sub-Saharan Africa on ART [11], significantly reducing morbidity and mortality due to HIV [1, 12]. The increase in ART coverage has also contributed to prevention, as individuals with suppressed viral load do not transmit the virus to others [13]. However, the percentage of people living with HIV who are virally suppressed is not yet sufficient to achieve epidemic control at the population level, and it will be necessary to intensify other prevention efforts as well [14].

Since 2012, the world has had access to another bio-behavioral prevention technology to help achieve epidemic control through the use of tenofovir-based pre-exposure prophylaxis (PrEP) [15–17]. Clinical trials have found that PrEP is highly effective in reducing risk of HIV acquisition across types of exposure, gender, PrEP regimens, and dosing schemes [18], and attention has turned to questions of how to optimize PrEP implementation [19–22]. There are now dozens of implementation research and demonstration projects either planned or underway across SSA and in multiple populations [19, 23]. The results of these projects will undoubtedly be helpful in moving the implementation field forward; however, many of them are not scheduled to be completed for several years. Meanwhile, in order to reduce new infections by 75% in 2020 as compared to 2010, UNAIDS has called for a rapid acceleration of PrEP delivery to reach 3,000,000 people as one of the five pillars of combination prevention [24].

A lack of formal implementation research results need not necessarily stymie the scale-up of PrEP delivery. In fact, the linear approach typically advocated by academics that moves from efficacy to effectiveness under controlled conditions to scaling to population level may not necessarily be ideal [25••, 26]. The characteristics that cause an intervention to be successful in efficacy or effectiveness research studies (e.g., intensive, supplementary staffing, highly standardized, tightly monitored) are fundamentally different from, and often at odds with, programs that succeed in population-based effectiveness settings (e.g., having broad appeal, being adaptable for both participants and intervention agents) [26]. In scaling-up PrEP delivery, as in ART delivery, the interconnectedness and dynamic interactions of actors closely resemble the characteristics of complex adaptive systems, wherein as delivery of the intervention progresses, population perceptions of the intervention change (both those receiving and those providing) which further provide different optimal ways of delivering the intervention [25••, 27–29]. In this environment, a “learning by doing” approach enables the context-specific adaptations to emerge [30, 31].

Given the current lack of evidence available for PrEP delivery at scale in SSA, how can we go about “doing” in the most effective way? Literature from psychology, cognitive, political and computer science, law and business explicitly describes the power of analogical reasoning for learning, decision making, and strategy development [32–36]. Though the global health literature is much less explicit in their use, drawing timely and accurate analogies between the scale-up of ART services and current efforts to scale-up PrEP could significantly increase the rate at which the highest priority populations have access to this important HIV prevention method [37]. The purpose of this review article is to summarize “lessons learned” during 15 years of scaling-up ART in SSA and to describe how they may or may not apply to PrEP scale-up. The hope is that such systematic comparisons will lead to fast-tracking safe and effective delivery of PrEP at scale so that its HIV prevention potential is soon realized at the population level. Key lessons from ART scale-up which are immediately applicable to PrEP delivery are discussed below and summarized in Table 1.

**Table 1:** Key lessons learned from scaling up ART in SSA and potential applications to the scale-up of PrEP

Lessons learned from ART delivery scale-up	Application to PrEP delivery scale-up	Additional considerations for PrEP
Scaled implementation required streamlining and minimizing laboratory tests. Symptom-directed approaches to laboratory monitoring of safety were adopted to reduce burden and costs to health system and clients.	Target population for PrEP are young and healthy with less likelihood of comorbid conditions than those on ART Simplifying requirements for laboratory monitoring will safely reduce barriers and costs to health system and clients.	Current safety and HIV epidemiologic data suggest HIV testing may be the only essential component of minimal lab safety package. Studies are needed to evaluate the safety of HIV testing strategies for PrEP users, including moving follow-up HIV testing from clinical settings to home-based HIV self-testing
Task-shifting from doctors to nurses was proven safe and dramatically increased access to ART.	PrEP is much less complicated and better tolerated than ART. Shifting PrEP prescribing authority from NIMART nurses to other health cadres will increase PrEP access.	Nurses working in family planning, maternal child health, anti-natal, and STI clinics are well-placed to provide PrEP alongside other reproductive health services. Models shifting PrEP delivery from nurses to community health workers and pharmacists should be developed, implemented and assessed.
Differentiated models of ART service delivery (including non-clinic-based services) have been essential to reducing burden on the health system and increasing access to services for those in need.	Differentiated PrEP service delivery models, (including non-clinic-based services) will be even more important to achieve PrEP delivery at scale as most clients will be healthy and not seeking other clinical services.	Non-clinic-based service delivery models (e.g. pharmacies, community points, tele-health/−medicine, private sector) used elsewhere should be tried in African settings. The education and social sectors should be engaged in demand and awareness creation, identification of potential users.
Overly cautious adherence preparation for individual clients created barriers to uptake and a “Test and Start” approach has been adopted. A range of adherence support strategies have been implemented.	Minimize emphasis on ‘willingness to adhere’ as requirements to start and re-start PrEP. Individual and public health concerns for interrupted adherence are importantly different for PrEP compared to ART.	PrEP is not intended for lifelong use but rather periods of risk. Viral mutations from interrupted adherence will be very rare. PrEP delivery requires a shift in thinking around ‘successful’ adherence at the individual and population level compared to adherence in ART delivery. Family planning may offer a more useful frame of reference for thinking about successful PrEP adherence.
Lowered age of consent and explicit policies and guidelines were necessary to reduce barriers for adolescent access to ART.	Lowered age of consent and explicit policies and guidelines will be necessary to reduce barriers for adolescent access to PrEP.	Adolescent girls at especially high risk of HIV may also be those for whom parental consent is a significant barrier. Consider explicitly linking lowered age of consent to related services that may be accessed alongside PrEP (e.g., HIV testing, family planning, mental health, ART, post-violence).
Stigma has been a barrier to HIV testing and treatment. Campaigns targeting “high risk populations” may have inadvertently contributed to stigmatizing people living with HIV as well as the sexual behavior through which their HIV was acquired.	The same stigma associated with HIV is also a barrier for PrEP uptake and persistence. Reduce emphasis on ‘risk’ and ‘risky’ behaviors in messaging for PrEP.	Shift public health messaging about PrEP from an emphasis on “risky behavior” to sex-positive, empowering language. Invest in strategies to increase community awareness to normalize pill taking for prevention.

## Minimize Laboratory Tests

Clinical trials and demonstration projects proving the efficiency and effectiveness of bio-medical interventions typically include an array of laboratory tests both to monitor patient safety as well as to provide data for research publications. Once the intervention has been proven safe and implementation possible, a more minimalist approach to laboratory monitoring must be considered to ensure scale-up is feasible. In high-income settings, ART patients often receive an array of laboratory tests on a quarterly basis, including viral load, CD4 counts, liver and renal function tests, and whole-blood counts [38]. However, in SSA, it would not have been possible to implement this protocol widely due to weaker laboratory infrastructure and costs. Not wanting limited laboratory infrastructure to unduly restrict access to ART, the WHO significantly simplified its HIV treatment and laboratory monitoring guidelines over the past two decades. In 2002, WHO guidance recommended a broad range of laboratory requirements for ART, prioritized in four categories: absolute minimum, basic recommended, desirable, and optional tests [39]. Subsequent WHO guidance has progressively emphasized a symptom-directed approach to laboratory monitoring of safety and toxicity for most ARV regimens, including for TDF-containing regimes that is part of the current PrEP formulation [15, 40]. Although empiric evidence on utility and frequency of laboratory monitoring is limited, one randomized trial which compared laboratory and clinical monitoring to clinically driven monitoring in Uganda and Zimbabwe found that ART could be given safely without routine laboratory tests to save more lives in Africa [41].

In thinking about PrEP delivery, policy makers and program implementers should be similarly pragmatic and dramatically simplify laboratory requirements in order to reduce barriers to PrEP uptake. The WHO 2017 guidance suggests a range of laboratory tests every 3–6 months for those on PrEP including for HIV, creatinine clearance, HBV, HCV, STIs, and pregnancy [42]. However, the majority of persons for whom PrEP is most useful are likely to find it challenging to obtain these laboratory tests this frequently in public health facilities and health providers will be less likely to embrace PrEP as a feasible HIV prevention intervention if they fear its safe implementation requires frequent laboratory monitoring. TDF-based PrEP is demonstrably very safe, with the most frequently reported side effect being gastro-intestinal symptoms which are normally self-limiting within 1–2 weeks of PrEP initiation [16, 43]. The risk of severe toxicities, including kidney injury and decrease in bone mineral density which were the initial main concern for PrEP, have been shown to be rare and limited both in frequency (< 2%) and magnitude [44–46]. The specific components of the PrEP minimal safety package may evolve over time with the emergence of new testing technologies and will vary across contexts, depending on such things as cost, infrastructure, and treatment availability. For example, testing for hepatitis B or C as a component of routine PrEP delivery seems counterproductive in a setting where no treatment is available, where healthcare providers are overstretched, and health systems severely under-resourced. The favorable safety profile for PrEP coupled with the fact that PrEP users are mostly young healthy persons supports a pragmatic and minimalist approach for laboratory testing in PrEP roll-out. For example, the Kenya guidelines for PrEP require HIV testing but are permissive of initiating and continuing PrEP without kidney function and hepatitis B testing if laboratory testing is not available [47]. Formal implementation science studies could more formally test the comparative cost effectiveness of various models, including no testing or only annual kidney safety monitoring with targeted testing for those with known risk factors for kidney injury.

Regular HIV testing is an essential component of PrEP delivery necessary to reduce the risk of antiretroviral resistance if HIV infection is present prior to PrEP, in which case PrEP will not be started, or occurs while prescribed PrEP, in which case it will be discontinued. Current guidelines recommend quarterly HIV testing for individuals on PrEP, operationalized mostly with provider-led clinic-based testing. Incorporating novel HIV testing technologies like HIV self-testing could result in greater implementation efficiency (reducing clinic visits and saving staffing and participant costs) in already overburdened health systems while maintaining fidelity of PrEP delivery. Ongoing studies experimenting a hybrid approach with HIV self-testing to replace quarterly clinic-based HIV testing with six-monthly clinic-based and quarterly HIV self-testing (Clinicaltrials.gov: NCT03593629) will help inform the safety and utility of this approach.

## Task Shifting

Early programs of ART delivery in sub-Saharan Africa were physician intensive. Several years into the launch of PEFPAR, it became clear that bringing ART delivery to scale would require changing that model to enable nurses to care for and prescribe ART. Multiple countries started training nurses in nurse initiation and management of ART (NIMART), and by 2010, there was extensive evidence as to the safety, feasibility, and desirability of nurses prescribing ART [48]. Evidence showed no difference in patient outcomes (virologic failure, mortality, toxicity failure) between physician-led and nurse-led initiation of ART and management of HIV care [49] and several studies showed improved patient satisfaction in non-physician initiated and managed care [50, 51]. The accumulation of this formal evidence eventually led to official policy changes about who could prescribe ART.

Because PrEP is an antiretroviral medication, many projects in Africa have identified HIV physicians, clinical officers, and NIMART nurses as the ones who should prescribe PrEP and those most likely to become experts in its delivery. Whereas this may be the most efficient use of human resources in some contexts, for example PrEP delivery for sero-discordant couples at ART clinics, too closely linking PrEP with ART prescription and management could discourage non-ART health providers from prescribing and managing PrEP, either formally through regulatory requirements or informally through assumptions that PrEP is as complicated as ART. Unnecessary restriction of PrEP prescription and refill authority will significantly constrain the reach of PrEP delivery and risk further over taxing ART service providers. Since PrEP is generally very well-tolerated and the risk of severe adverse events including kidney impairment are very rare [43, 52], moving quickly to PrEP provision by non-NIMART-trained nurses and other healthcare cadres should be strongly encouraged. Nurses working in family planning, maternal child health, anti-natal, STI, and other clinics where there is routine HIV testing would be very well-placed to implement rapid screening for and initiation and follow-up of PrEP clients [53]. Implementation research should focus on how best to motivate and engage healthcare workers to consider integration of PrEP provision into sexual and reproductive health and other health services. Pharmacists and community health workers working in community settings may better meet the needs of populations historically less likely to seek services at a health facility (see section below on differentiated service delivery for further discussion). Policy makers concerned with non-clinical specialists routinely providing refills and ongoing support for individuals wanting to take PrEP can look to the evidence from ART scale-up that demonstrates non-specialist medication delivery can be both safe and effective and rapidly move forward non-clinic-based approaches of PrEP delivery. Once implemented, programmatic data from these various approaches produced in different contexts as well as more intensively collected implementation research data can inform ongoing program refinement.

## Differentiated Models of Service Delivery

Public ART services in SSA were initially concentrated in clinics in major hospitals, which were very quickly inundated with patients. It soon became apparent that overly crowded ART clinics, as well as long distances between client homes and these centralized ART delivery points, threatened access to and retention in HIV care and treatment. In response, a range of service delivery models differentiated by the service frequency, location, intensity, provider, or some combination of these [54] have been implemented to decongest health facilities, bring ART closer to where clients work and live, and reduce the time burden and transaction costs of clients needing HIV treatment. Models have been tailored to respond to differences in clinical characteristics of clients (stable, unstable, co-morbidities), sub-population (men, women, children, key populations), and context (urban, rural, stability, epidemic type) [55•].

Innovations in ART differentiated service delivery have included (1) facility-based individual models, for example, where ART refill visits have been separated from clinical consultations so as to “fast-track” ART refills [56] and with appointment spacing extended from 3 to 6 months [57, 58]; (2) models where ART refills and some clinical consultations are provided to individuals outside of healthcare facilities, for example using drug distribution (CDDP) systems in Uganda [59, 60] or home delivery [61]; (3) group models where clients receive their ART refills in a group managed by a lay healthcare staff member, such as in adherence clubs in South Africa [62] and Kenya [63]; and (4) client-managed group models, where clients receive their ART refills in a group but this group is managed and run by clients themselves [56, 64]. Overall, the results of these efforts have shown improved rates of ART coverage and patient satisfaction [65, 66] and decreases in loss to follow to follow-up and mortality [67, 68].

The need for differentiated service delivery will be crucial for PrEP scale-up as well. Individuals who are not feeling sick and have no HIV diagnosis may be even less inclined than those living with HIV to wait in long health lines and travel to special health facilities. Some of the populations most at risk for HIV (e.g., MSM, CSW, and AGYW) may be especially disinclined to access prevention services at health facilities [19]. Implementers should consider how to modify and scale PrEP service delivery models centered on these and other unique needs of PrEP clients. Recent innovations from outside SSA provide promising models for scale including PrEP in community pharmacy settings [69] and tele-medicine-assisted models in both the public and private health sectors [70]. Early learning from these models suggests PrEP service delivery outside of clinical settings as well as outside the public health sector is feasible and acceptable. Implementation approaches should further consider intersectoral engagement in PrEP service delivery and the potential contribution of education, social, and other sectors in generating demand and awareness, identifying potential users, and as possible delivery points for PrEP provision and follow-up services.

A challenge for non-facility-based PrEP delivery not shared by ART services is the need for repeat HIV testing to confirm ongoing HIV-negative status and hence eligibility for PrEP continuation. As discussed above, further implementation research is needed on improving correct HIV diagnosis at point of service locations with testing algorithms that can be performed rapidly and effectively without expensive equipment or specialized laboratory technicians [71]. HIV self-tests have significant potential in this regard, although current challenges exist with high self-test kit costs and reliability of results due to user performance errors [72]. Increasing the reliability, adoption, and adaptation of HIV-self testing will be key to ensuring feasibility of decentralized and community-based PrEP services.

## Adherence Readiness Concerns and Scaled Uptake

Adherence is key for the effectiveness of both ART and PrEP. Since the start of ART scale-up in SSA, adherence has justifiably been a central concern as sub-optimal adherence can lead to development of drug-resistant mutations both at the individual and population level [73]. Concerns about individuals being able to take their medication regularly underlay the cautious approach to ART initiation in the early years of scale-up, wherein attending several adherence counseling sessions to demonstrate readiness and commitment to take treatment regularly and correctly was required before ART medication could be prescribed [74, 75]. However, substantial attrition between testing and treatment occurred under this model. In a systematic review of retention outcomes in HIV care between testing and treatment in SSA, Rosen et al. [76] estimated that less than one-third of patients testing positive for HIV but not eligible for ART when diagnosed were retained in pre-ART care continuously, including those patients who did not return for their initial CD4 count results and those in the preparatory phases of ART initiation. After a decade of scale-up, the perceived benefit of ensuring adherence readiness through multiple counseling sessions was outweighed by concerns of lost opportunity to link HIV+ individuals to care with more immediate start of ART; the WHO recommended a “Test and Start” approach, wherein individuals diagnosed with HIV are started on ART immediately [15]. The lesson we can draw from this for PrEP uptake and continuation is to make it as easy as possible for those who request PrEP to get it. Delaying or refusing PrEP due to concerns about willingness to adhere is likely to have a deterrent effect on individuals seeking prevention medication.

In clinical trials and demonstration projects to date, monitoring PrEP adherence has been essential to determining whether it was truly effective. However, transitioning from research to implementation at scale necessitates thinking about PrEP adherence differently. Although a range of ART adherence interventions (e.g., SMS, adherence clubs, economic incentives, and intensified counseling approaches) [77] may be applicable to supporting PrEP clients, PrEP adherence requirements and concerns are fundamentally different from those of ART along several key dimensions. These differences need to be carefully considered as countries calibrate their level of investment in adherence strategies for routine PrEP delivery. First, the public health risk of individual non-adherence is much less for PrEP than for ART. Sub-optimal adherence to PrEP may result in an individual acquiring HIV but evidence suggests instances of viral mutation will be rare [78–81]. Second, unlike ART, PrEP is not intended for lifelong use, but only for seasons of potential HIV exposure. “Prevention-effective PrEP adherence” requires the use of PrEP only during periods of risk exposure and when alternative prevention tools, such as condoms or the use of ART to achieve viral suppression by a known HIV-infected partner, are not used [82]. As such, discontinuation of PrEP has different implications for definitions of program success than does the discontinuation of ART. In thinking about what programmatic outcomes constitute successful adherence to PrEP in routine settings, family planning may offer a useful frame of reference. Just as family planning programs have prevented many unplanned pregnancies despite imperfect risk assessment and use of contraception, more HIV will be prevented with PrEP than without it [82]. Further policy discussions, implementation research, and modeling are necessary to generate consensus on acceptable PrEP adherence at the population level.

## Parental Consent and PrEP Access for Adolescents

During the scale-up of ART, requiring parental consent for an HIV test was recognized as a barrier for adolescents who wanted to avoid telling parents about their sexual activity [83–85]. In response, many African countries formally lowered the age of consent for HIV testing from aged 18 to as low as 12 years [85]. However, only one country (Madagascar) also explicitly allows ART treatment without parental consent [84]. Resolving issues surrounding parental consent both for HIV testing and PrEP medication will be equally important for scaling PrEP delivery to this demographic, especially as adolescent girls in SSA are a particularly vulnerable age group for HIV acquisition [20]. Evidence from ART scale-up suggests that where age of consent guidance is limited or vague or policies not well understood, health providers are frequently reluctant to provide services or impose age restrictions based on their own values and judgments [86, 87]. Studies on the provision of HIV counseling, testing, and treatment [84, 85, 87] as well as of family planning services [88, 89] suggest that clear and congruent laws, policies, and guidelines permitting independent consent to both HIV testing and medication will be important to ensure PrEP access and prevention impact across this population. Countries might consider explicitly linking lowered age of consent to related services that may be accessed alongside PrEP (e.g., HIV testing, family planning, mental health, ART, post-violence).

## Reduce Stigma and Normalize Use

From its early days, HIV has been stigmatized as a disease acquired through undesirable (multiple sexual partners) or illegal sexual behavior (as in the case of commercial sex workers and MSM). Targeting high-risk categories in public campaigns can unintentionally fuel stigma and discourage individuals from testing for HIV, because they do not identify as or want to be associated with one of these groups [37, 90]. Stigma associated with HIV has also impacted the way HCW interact with the population, further reducing incentives for individuals to be tested for HIV and for those living with HIV to seek care and treatment [91]. Over the course of the response to the HIV epidemic, HIV testing has become increasingly routinized [92], and there is some evidence that societal and self-stigma has decreased as ART coverage has increased [93], although the evidence is mixed [94].

Likewise, strategies to encourage PrEP adoption and adherence must proactively consider the role of stigma in uptake and delivery. In several contexts, PrEP stigma has been identified as the most significant community level barrier to PrEP uptake and adherence [95–97]. PrEP programs, policy and research, must be careful not to reinforce or amplify this stigma [98•]. Currently, most PrEP messaging is for individuals at “very high risk*.*” The extent to which PrEP users come to be perceived by the community (including health care workers) as “high risk” or “promiscuous” could greatly undermine PrEP uptake [19, 20]. Evidence suggests that societal stigma associated with PrEP will influence individuals to underestimate their risk for HIV (and hence eligibility for PrEP) as they seek to reduce their association with these stigmatized groups [37]. Changing PrEP messaging to make it more engaging, sex positive, and intimacy focused will greatly increase the likelihood that PrEP uptake will be achieved at the scale necessary for population level prevention impact [20, 99]. Changing PrEP messaging to focus on those wanting to take responsibility for their sexual health and to reduce concerns around HIV infection constructs PrEP as a socially desirable behavior and may even promote condom use by promoting what has been termed a “preventionist identity” [98].

## Conclusion

With broad dissemination and uptake, PrEP has significant potential to reduce population level HIV incidence. Just as PrEP can only prevent HIV at the individual level if taken effectively, PrEP can only significantly impact epidemic control if it is scaled rapidly and effectively to reach populations experiencing high levels of HIV incidence. Results of ongoing and future implementation research will be instrumental in finetuning PrEP delivery to maximize benefit while minimizing costs. Meanwhile, rapid introduction and scaling of PrEP programs to meet global targets can be facilitated by borrowing evidence from the scale-up of ART and using programmatic data to rapidly assess effectiveness in real time and modify delivery models in different contexts. Experience with ART service delivery suggests that scaling will require both simplifying and diversifying approaches so that PrEP delivery is feasible for health systems and accessible to the various populations for whom PrEP is the best prevention tool. As countries with high HIV prevalence make progress in implementing a public health approach to PrEP, the context of PrEP delivery will evolve. Developing streamlined data collection systems will be essential to capture the dynamic interaction between context and delivery and to ensure ongoing “learning by doing” occurs.
