# Correction to: Scaling-up PrEP Delivery in Sub-Saharan Africa: What Can We Learn from the Scale-up of ART?

**DOI:** 10.1007/s11904-019-00443-8

**Published:** 2019-04-22

**Authors:** Gabrielle O’Malley, Gena Barnabee, Kenneth Mugwanya

**Affiliations:** Department of Global Health, University of Washington, Seattle, WA USA

**Correction to: Curr HIV/AIDS Rep (2019) 16:141–150**

10.1007/s11904-019-00437-6

The article “Scaling-up PrEP Delivery in Sub-Saharan Africa: What Can We Learn from the Scale-up of ART?”, written by Gabrielle O’Malley, Gena Barnabee and Kenneth Mugwanya, was originally published electronically on the publisher’s internet portal (currently SpringerLink) on 22 February 2019 without open access.

With the author(s)’ decision to opt for Open Choice the copyright of the article changed on April 2019 to © The Author(s) 2019 and the article is forthwith distributed under the terms of the Creative Commons Attribution 4.0 International License (http://creativecommons.org/licenses/by/4.0/), which permits use, duplication, adaptation, distribution and reproduction in any medium or format, as long as you give appropriate credit to the original author(s) and the source, provide a link to the Creative Commons license and indicate if changes were made.

The original article has been corrected.

